# Activation of *FGFR* genes by genetic and epigenetic alterations in uterine leiomyomas

**DOI:** 10.1038/s44276-025-00127-4

**Published:** 2025-02-27

**Authors:** Vilja Jokinen, Aurora Taira, Åsa Kolterud, Isa Ahlgren, Kimmo Palin, Riku Katainen, Maritta Räisänen, Eevi Kaasinen, Sini Ilves, Anniina Raitila, Helena Kopp Kallner, Emma Siili, Ralf Bützow, Oskari Heikinheimo, Annukka Pasanen, Auli Karhu, Niko Välimäki, Lauri A. Aaltonen

**Affiliations:** 1 Department of Medical and Clinical Genetics, University of Helsinki, Helsinki, Finland; 2 Applied Tumor Genomics Research Program, Research Programs Unit, University of Helsinki, Helsinki, Finland; 3 Department of Medicine Huddinge, Division of Biosciences and Nutrition, Karolinska Institutet, Huddinge, Sweden; 4 iCAN Digital Precision Cancer Medicine Flagship, University of Helsinki, Helsinki, Finland; 5 Department of Clinical Sciences, Danderyd Hospital, Karolinska Institutet, Stockholm, Sweden; 6 Department of Obstetrics and Gynecology, Danderyd Hospital, Stockholm, Sweden; 7 Department of Pathology, University of Helsinki and Helsinki University Hospital, Helsinki, Finland; 8 Department of Obstetrics and Gynecology, University of Helsinki and Helsinki University Hospital, Helsinki, Finland

## Abstract

**Background:**

*Fibroblast growth factor 1-4 (FGFR1-4)* are well-known oncogenic drivers in many cancer types. Here, we studied the role of FGFRs in uterine leiomyoma (UL) that is a benign neoplasm arising from the myometrium and the most common tumour in women. Although ULs can be classified to molecular subtypes based on genetic drivers, potential secondary drivers are not well characterised.

**Methods:**

We performed mutation analysis of RNA-sequencing data of ULs, followed by screening of *FGFR* alterations in our Finnish (*n* = 2677) and Swedish (*n* = 372) UL collections, utilising Sanger-, next-generation and Nanopore sequencing and SNP array data. The role of *FGFR* genes in UL predisposition was examined by GWAS.

**Results:**

We identified FGFR activation in a subset of ULs on both genetic and epigenetic levels. In addition to single-nucleotide mutations in *FGFR1/2*, we detected an *FGFR2-ERC1* fusion gene, *FGFR1* gains and hypomethylation of regulatory regions of *FGFR2/3*. *FGFR* alterations were enriched in molecularly similar HMGA2, HMGA1 and PLAG1 UL subtypes. We also unveil a UL predisposing variant upstream of *FGFR4* associated with increased expression in both normal myometrium and ULs.

**Conclusions:**

Our results establish the role of FGFR signalling in the genesis of UL.

## Background

Uterine leiomyomas (ULs) are benign neoplasms of the myometrium. Up to 70% of women develop ULs by the age of 50, and approximately one in four develop symptoms, including abnormal uterine bleeding, pelvic pain and infertility [1, 2]. Currently, the only curative treatment options are invasive, and one third or even half of all hysterectomies are conducted for UL patients [3]. Due to the high prevalence and lack of non-invasive treatments, UL related costs have been estimated to exceed the combined cost of colorectal and breast cancer in the United States [4].

Multiple molecular subtypes of ULs with typically mutually exclusive genetic drivers have been discovered: hotspot mutations of *MED12*, overexpression of HMGA2, biallelic inactivation of *FH*, SRCAP complex genes involved in histone H2A.Z loading and a group of genes affecting neddylation of Cullin 3-RING E3 ligase [5–10]. In addition, HMGA1 and PLAG1 overexpression sometimes due to chromosomal translocation and COL4A5-COL4A6 deletions through *IRS4* overexpression drive UL tumourigenesis [11, 12]. The genetic driver alteration remains undiscovered for about 10% of ULs. Possible secondary genetic driver alterations are not well understood.

Fibroblast growth factor (FGF)/FGF receptor (FGFR) signalling plays a crucial role in the regulation of cell differentiation, proliferation and apoptosis in multiple cell types both in embryonic development and in adults [13]. FGFRs are transmembrane tyrosine kinase receptors that mediate the FGF signalling cues to the cytosol, activating RAS/MAPK, PI3K/AKT and DAG/PKC signalling pathways [13].

In humans FGFRs are encoded by four genes, *FGFR1-4*. Activating mutations of *FGFR* genes, including point mutations, chromosomal rearrangements, fusion genes and copy number amplifications are seen in 5–10% of all human cancers [14]. *FGFR1* mutations tend to aggregate codons encoding two hotspot amino acids, asparagine 546 and lysine 656, in the tyrosine kinase domain [15]. *FGFR2* mutations, that are common in endometrial, breast and lung cancer, are seen also in transmembrane and extracellular domains [15, 16]. *FGFR1* amplifications are very common in many cancer types, including lung and breast cancer, whereas *FGFR2-4* amplifications are less frequent, yet occurring e.g. in gastric and breast cancer [14, 16]. *FGFR1-3* fusion genes have been reported with multiple partner genes in many malignancies, including glioblastoma, breast and lung cancer [16].

Previous studies have suggested that myometrial and endometrial *FGFR1* expression levels vary in different stages of menstrual cycle but this fluctuation is disturbed in ULs and endometrium of UL patients, suggesting that *FGFR1* might play a role in the regulation of menstrual cycle and in the tumourigenesis of Uls [17, 18]. In addition to this, it has been shown that *FGFR1* gain can lead to *FGFR1* overexpression in Uls [19]. However, such changes are somewhat unspecific and activating *FGFR* point mutations have not to our knowledge been reported. Here, we performed analysis of multiple layers of sequencing data and SNP array data showing that activating mutations in *FGFR* genes do indeed occur in a subset of ULs, strongly supporting the role of FGFR signalling in the genesis of ULs. Furthermore, examination of genome-wide association studies (GWAS) revealed a possible role of *FGFR4* in genetic predisposition to ULs.

## Methods

### Sample collection

**Fig. 1:**
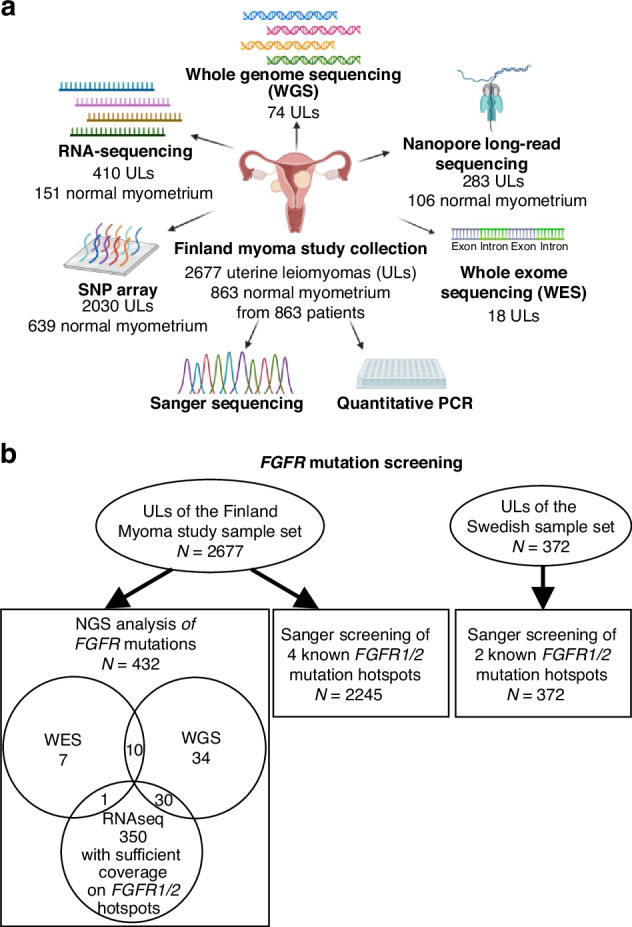
Summary of methods. **a** All methods used in this study to detect *FGFR* gene alterations in the Finland Myoma Study collection (*n* = 2677). **b** Data types utilised to screen *FGFR* mutations in the Finnish and Swedish UL sample sets.

The current Finland Myoma Study sample set comprises 2677 UL samples from 863 individuals, and the corresponding normal myometrium samples for each patient (Fig. 1a, Table S1). This sample set largely overlaps the previously published sample set [9]. UL subtypes were defined as described in Supplementary Material and Methods (Fig. S1). In addition, the Swedish sample set of 372 ULs from 147 patients was collected from hysterectomy and myomectomy patients at Danderyd Hospital. All samples were collected after a written informed consent.

### DNA and RNA extractions

For the Sanger sequencing, Nanopore long-read sequencing and SNP array, DNA was extracted from the fresh frozen tissues using QIAamp FAST DNA Tissue Kit (Qiagen, Hilden, Germany) or FastDNA Kit (MP Biomedicals LLC, Santa Ana, California, USA). For the previously published whole-exome sequencing (WES) and whole-genome sequencing (WGS) data, samples were processed as previously described [5, 20, 21]. For RNA-extractions, we used TRIzol Reagent (Invitrogen, Waltham, Massachusetts, USA), RNase-free Dnase (Qiagen) and RNeasy MiniElute clean-up kit (Qiagen). RNA was converted to complementary DNA (cDNA) by M-MLV Reverse Transcriptase (Promega, Madison, Wisconsin, USA) following standard reverse transcription procedures [9].

### RNA-sequencing

RNA-sequencing (RNA-seq) data were generated from altogether 410 ULs and 151 corresponding normal myometrium samples from 324 patients, in part overlapping with a previously published RNA-seq dataset [9]. To this dataset, representative samples of MED12, FH and HMGA2 subtypes were selected but ULs without driver mutations identified were the primary focus. The RNA libraries were prepared using Illumina TrueSeq Stranded Total RNA with Ribo-Zero human kit (Illumina, San Diego, California, US) following the manufacturer’s instructions. RNA-seq was performed with Illumina HiSeq 2500 in SciLife for 124 samples and with NovaSeq 6000 in Macrogen for 437 samples [9].

We used Trim Galore v.0.5.0 for the quality and adaptor trimming. The reads were aligned by Salmon v1.8.0 to the default salmon transcriptome reference (accessed on May 23, 2022) using the partial selective alignment method. DESeq2 v1.34.0 and limma v3.50.0 were used to produce variance stabilised, batch-corrected gene expression values [22, 23]. Unsupervised hierarchical clustering was done utilising Ward linkage and 1-correlation distance across *n* = 1355 (5%) most variable genes. The analysis of differentially expressed genes between sample groups was done using DESeq2 v1.34.0, and log-fold-change shrinkage was done using the option type = ‘apeglm’ [22]. Only genes with the coverage of at least 10 reads in at least five samples were analysed (28,199 genes), and the sequencing batch was given as a confounder. For the multiple test correction, DESeq2 uses Benjamini-Hochberg method to produce adjusted *p* values (padj).

For the mutation calling, STAR v2.7.9a 2-PASS alignment was performed to align the reads to the reference genome GRCh38 [24]. To prepare the data for mutation calling, Genome Analysis Toolkit (GATK) v4.2.4.1 tools MarkDuplicates, SplitNCigarReads, BaseRecalibrator and ApplyBQSR were used [25]. The 1000 Genomes Project, gnomAD, and Mills SNVs and indels, and GATK’s indels were given as known variable sites for the GATK BaseRecalibrator. We used GATK Mutect2 for the variant calling with the options --dont-use-soft-clipped-bases and --pcr-indel-model AGGRESSIVE. The panel of normals was created by GATK GenomicsDBImport and GATK CreateSomaticPanelOfNormals from 40 random normal myometrium samples. Mutations were filtered against the panel of normals, with the maximum median fragment length difference of 20,000 and based on strand bias, and against the Haplotypecaller variant calls of the corresponding normals. Using BasePlayer v2, we annotated variants with the MANE transcriptome [26, 27]. Variants were further filtered against gnomAD exome v2.1.1 and genome v3.0 databases utilising ANNOVAR [28, 29]. Minimum coverage of 10 and minimum allelic fraction of 1/3 were required. The exact same deletions seen in at least two UL samples were filtered out. The InterPro database was used to obtain the domain information [30].

Fusion genes were identified using the FusionCatcher (v1.33) software and default FusionCatcher database (hg38, Ensembl v102) [31]. The resulting fusion calls were filtered to a minimum x3 coverage based on the ‘spanning unique reads’ and ‘spanning pairs’ values. All in-frame fusions involving *FGFR* genes were then inspected for further supporting evidence in Nanopore DNA sequencing data.

### *FGFR1/2* mutation screening and validation

The results from RNA-seq mutation calling led us to screen for *FGFR* mutations in the whole Finnish sample set of 2677 ULs, utilising all the readily available sequencing data as well as Sanger sequencing of samples where no data was available. For the samples with NGS data (*n* = 432), mutation calls of *FGFR* from RNA-seq, WGS and WES data were used (Fig. 1b, Table S1). The minimum read depth of 15 at the three most common *FGFR1* hotspots and the four most common *FGFR2* hotspots according to the Catalogue Of Somatic Mutations In Cancer (COSMIC) was required with RNA-seq data [15]. WGS and WES data were processed as previously described [20, 21]. Germline variants were filtered out against gnomAD exome v2.1.1 and genome v3.0 databases utilising ANNOVAR [28, 29]. Liftover from GRCh37 to GRCh38 was performed for the variant calls of WES and CompleteGenomics WGS data using GATK LiftoverVcf [25]. The support of at least three reads was required, and WES and Illumina WGS variants were annotated with the MANE transcriptome using BasePlayer v2 [26, 27].

**Fig. 2:**
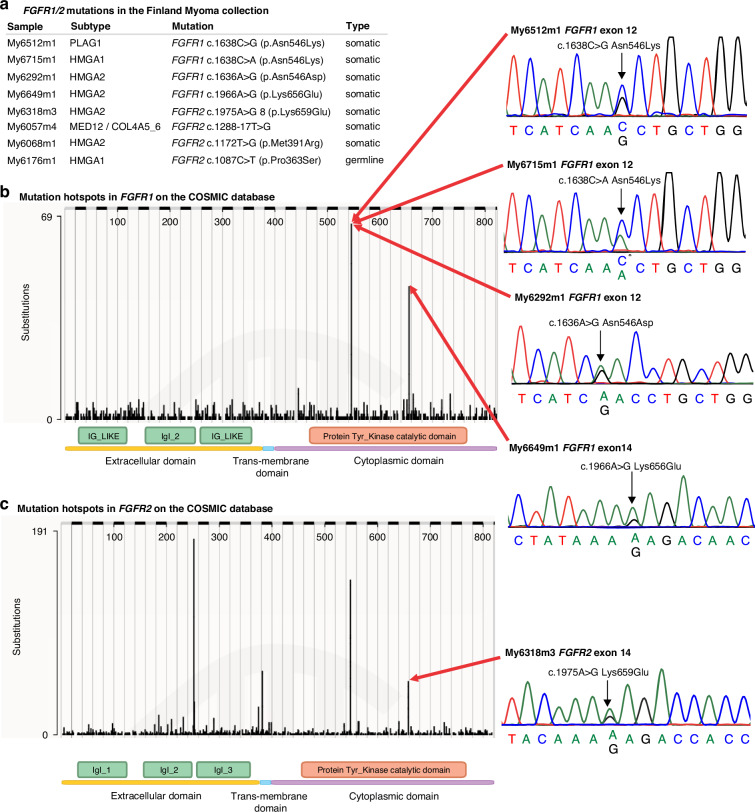
Single-nucleotide mutations in *FGFR1/2.* **a** Altogether eight single-nucleotide mutations were found in *FGFR1/2* in the Finland Myoma Study Collection. **b**, **c** Distribution of single-nucleotide mutations in *FGFR1/2* in the COSMIC database of cancer mutations and the Sanger validated mutations in *FGFR1/2* mutation hotspots found in our UL sample set. The domain information was obtained from the InterPro database [30].

Sanger sequencing covering areas encoding the known cancer hotspot codons 546 encoding asparagine in *FGFR1* and 659 encoding lysine in *FGFR2* was performed in samples with no NGS sequencing data available (*n* = 2245), and for the Swedish UL sample set (*n* = 372). For the Finnish sample set, the areas covering the known cancer hotspot codons 549 encoding asparagine in *FGFR2* and codon 656 encoding lysine in *FGFR1* were also screened (see Fig. 2). Variants detected in NGS data were validated by Sanger sequencing. The PCR amplification and Sanger sequencing were performed as previously described [5].

Possible enrichment of *FGFR* mutations to specific UL subtypes was examined with Fisher’s exact test. Only one UL per subtype per patient was included in the analysis, and ULs with more than one known subtype were excluded.

### Long-read sequencing

The Nanopore long-read sequencing data from altogether 283 ULs and 106 normal myometrium samples, in part previously published [9], were generated and processed as described in Supplementary Material and Methods. Methylation values were calculated for each CpG as the proportion of methylated reads from all reads. A read coverage of 3-75 reads was required for each CpG utilised in the analysis. Average methylation at the promoter region (1000 bp upstream from transcription start site [TSS]) was defined as the average methylation of the CpGs overlapping the promoter region. A minimum of 5 measured CpGs from the promoter was required for all samples. Locations for promoter regions were downloaded from UCSC using the Table Browser tool [32]. The linear effect due to the pore version was subtracted from the average promoter methylation. In addition, per CpG methylation values were collected for each *FGFR* gene 15Kbp upstream and downstream from the TSS. The geom_smooth()-function from R-package ggplot2 was used for plotting the regional methylation levels [33].

Single-nucleotide variants were called using Longshot (v.0.4.3). Variants seen with significant strand-bias or with excessive alternative allele base-calling (support of four or more reads across at least 40 samples) were filtered out as technical artefacts. Bad quality regions were defined in 250 bp windows if at least 10 normal samples showed coverage <5 with mapq>10 reads. Variants were filtered against the panel of normals formed by the normal myometrium samples using bcftools v.1.9 and lifted over to GRCh38 using GATK LiftoverVcf [25, 34]. Variants were further annotated with the MANE transcriptome and Alpha Missense predictions using BasePlayer v2 and filtered against gnomAD exome v2.1.1 and v4.0 and genome v3.0 and v4.0 databases [26, 27, 35]. The minimum of four mutant allele reads was required. The mutation calls were inspected for the mutation hotspots of *FGFR* genes based on COSMIC database [15].

Structural variants (SV) were called using cuddlySV v3.0.0 (https://github.com/kpalin/cuddlySV) [36, 37]. At least four supporting reads were required for each SV call. SVs seen in myomas of at least two different patients within 5 bp window were excluded as probable artefacts. Only SVs with a breakpoint within 1 Mbp from *FGFR* genes were included. For the intrachromosomal alterations, the minimum length of 350 bp was required. The last filtering step was performed by visual inspection of each SV call by Integrative Genomics Viewer [38].

### SNP array data

Altogether 2,030 ULs were genotyped with HumanOmni 2.5–8 (n = 88) or Illumina Infinium HumanCore-24 chips (*n* = 1,942). Allelic imbalance segments were identified as previously described [9]. Somatic copy number variation analysis was performed using ASCAT v3.1.2 [39] for Illumina Infinium HumanCore-24 samples excluding clonally related tumours as earlier described (*n* = 1922) [9]. Regions where the number of copies exceeded the predicted overall ploidy of the sample by over 20% were considered as a somatic gain.

### Quantitative PCR

*FGFR1* expression was assessed using real-time quantitative PCR (qPCR). We used TaqMan probes for *FGFR1* (Hs00241111_m1 and Hs00915142_m1) and the housekeeping gene *HPRT1* (Hs02800695_m1) with the 7500 Fast Real-Time PCR System (Thermo Fisher Scientific, Waltham, Massachusetts, US). The samples were analysed as triplicates. A *MED12*-mutated UL was used as a reference sample to compare the other samples against.

### Genome-wide association study

GWAS was compiled as a meta-analysis of three cohorts: FINNGEN (https://finngen.fi/, release 10, accessed June 2024) [40], UK Biobank (UKB, Application Number 80756, accessed June 2021) and Biobank Japan (BBJ, http://jenger.riken.jp/en/, accessed on Sept 2020). The FINNGEN cohort had a total of 34,422 UL cases and 195,888 female controls. The UKB cohort of white British women comprised a total of 18,014 UL cases and 202,535 female controls. The BBJ cohort comprised a total of 5,954 UL cases and 95,010 female controls. The phenotype definition, population stratification and genotype quality-control steps were described in our previous publication [9]. All cohort-wise summary statistics were precomputed with mixed model logistic regression (SAIGE); details are available at the sources listed above. The BBJ and UKB data were lifted over to GRCh38/hg38 coordinates (Picard LiftoverVcf). An inverse-variance weighted fixed effects meta-analysis (R package ‘meta’ v4.8-4) was applied to 6.3 million SNPs that were available from all three cohorts. Expression quantitative trait loci (eQTL) were calculated as an additive effect of the GWAS risk allele using DESeq2, while adjusting for patients’ age at hysterectomy, ancestry (principal component analysis of population structure) and RNA-seq batch.

## Results

### Somatic point mutations in *FGFR1* and *FGFR2*

In the mutation calling from RNA-seq data, we identified altogether nine genes that were mutated in at least four ULs with different mutations (Table S2). Five of these genes (*MED12*, *FH*, *ACTL6A*, *DMAP1*, *YEATS4*) were already known UL driver genes. One gene, *FGFR2*, showed a well-known cancer-related hotspot mutation p.Lys659Glu [15] and three other mutations. The closer investigation of *FGFR1-4* genes revealed *FGFR1* mutations on the hotspot 546 encoding asparagine in three ULs (Fig. 2). These results led us to screen *FGFR* genes in the whole UL collection, and we detected another cancer related hotspot mutation *FGFR1* c.1966A>A/G p.Lys656Glu (Fig. 2, Table S3).

All *FGFR* mutations except for *FGFR2* p.Pro363Ser were validated somatic (Fig. 2, Table S3). For the mutations close to intron-exon boundaries *FGFR2* c.1288-17 T > G and p.Pro363Ser, also cDNA sequencing was performed but no splice effect was recognised.

### *FGFR2-ERC1* fusion gene and structural variants in *FGFR* genes

**Fig. 3:**
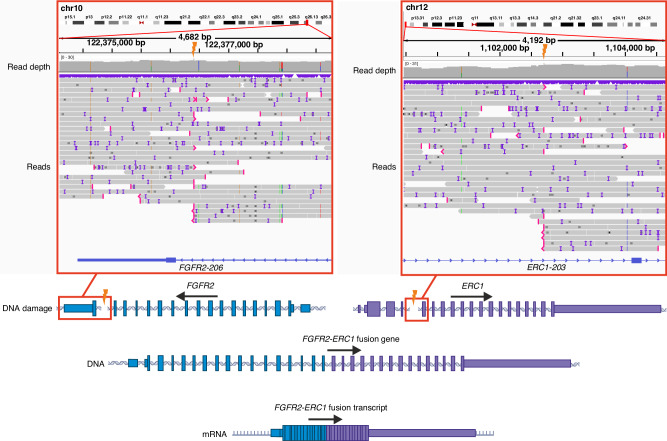
An in-frame *FGFR2-ERC1* fusion gene in one UL. The Nanopore DNA-sequencing data of UL sample My6006m1 is visualised on IGV on the two locations showing the breakpoints of the somatic translocation leading to the fusion gene. The DNA breaks of split reads shown with red display the breakpoints. In the resulting fusion gene, exons 1-17 of *FGFR2* are fused with exons 5-19 with *ERC1*.

In the RNA-seq dataset, an in-frame fusion gene of *FGFR2* was identified in one UL without any other known driver alterations (My6006m1). The first 17 exons of *FGFR2* were fused with exons 5-19 of *ERC1* (Fig. 3). This alteration was also confirmed by SV analysis of Nanopore long-read sequencing data. No in-frame fusion genes were found for the three other *FGFR* genes.

To detect possible FGFR-related SVs from the Nanopore data of 283 ULs, we looked for breakpoints at most 1Mbp from the *FGFR* genes and identified 18 *FGFR1* SVs in 5 ULs, 7 *FGFR2* SVs in 4 ULs, 20 *FGFR3* SVs in 14 ULs (from 12 patients), and 7 *FGFR4* SVs in 6 ULs (Table S4). In addition to the *FGFR2-ERC1* fusion, no intragenic breakpoints were found in *FGFR* genes. RNA-seq data was available for 27/28 of the ULs with *FGFR* SVs. However, only one of these ULs, My1004m1 without a known driver alteration, showed slightly higher *FGFR4* expression (Fig. S2.). This lesion had multiple SVs in chromosome 5, and the closest breakpoints from *FGFR4* were 491 Kbp downstream from *FGFR4*.

### Gains of chromosome 8 driving *FGFR1* overexpression

**Fig. 4:**
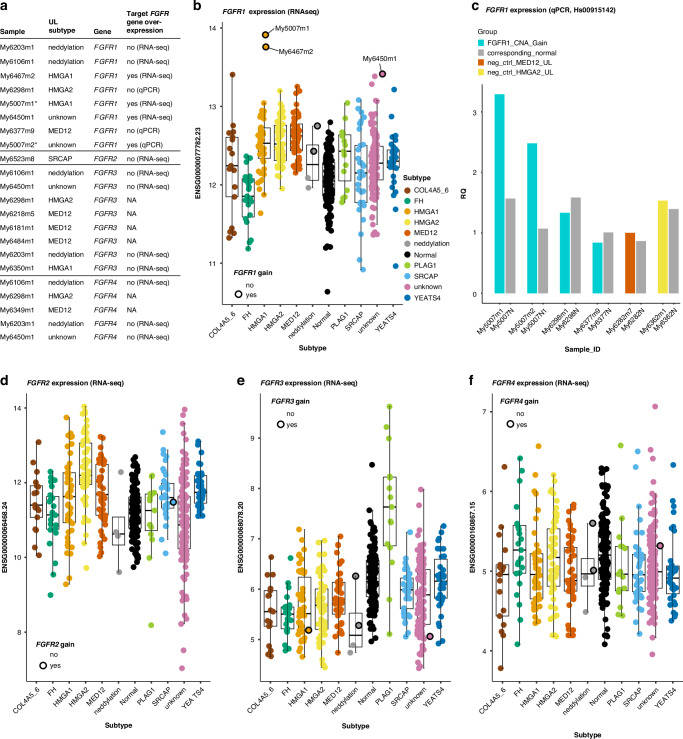
Chromosomal gains and expression of *FGFR* genes in ULs. **a** Chromosomal gains overlapping *FGFR* genes in ULs with SNP array data (*n* = 2030). **b** *FGFR1* expression levels in different UL subtypes and normal myometrium samples. ULs with chromosomal gains overlapping *FGFR1* are circled with black line. Of these, sample names are depicted for the cases that show clear overexpression. **c** *FGFR1* qPCR results for the ULs with an amplification of *FGFR1* but no RNA-seq data. My5007m1 was used as a positive control, and random MED12 and HMGA2 ULs (My6282m7, My6326m1) as negative controls. The MED12 UL was used as a reference sample for calculating the relative quantification (RQ) values. **d**–**f** *FGFR2-FGFR4* expression levels in different UL subtypes and normal myometrium samples. None of the ULs with gained regions overlapping these genes showed clear overexpression.

From the 2030 ULs with SNP array data, altogether eight ULs displayed chromosomal gain overlapping *FGFR1* (Fig. 4a, Table S5). RNA-seq data were available for five of these ULs, and three of them were the three most highly *FGFR1* expressing samples of the whole RNA-seq dataset (Fig. 4b). *FGFR1* expression levels were evaluated using qPCR for the samples with no RNA-seq data, and *FGFR1* overexpression was confirmed in one additional UL sample, My5007m2 (Fig. 4c). Thus, altogether 4/8 ULs with a gain overlapping *FGFR1* showed overexpression of *FGFR1* (Table S5). Two of these tumours, My5007m1 and My5007m2, were clonally related. The closer look on these four ULs revealed that tumours My5007m1, My5007m2, and My6450m1 had a whole chromosome 8 gain of both homologous chromosomes. UL My6467m2 had gained almost the whole chromosome 8 except for a small part of the p-arm (Fig. S3).

Large gained regions overlapping the other three *FGFR* genes were also seen in many ULs: one with *FGFR2*, eight with *FGFR3* and five with *FGFR4*. However, no overexpression of these genes was seen in these ULs with the available RNA-seq data (Table S5, Fig. 4d–f).

### Hypomethylation at *FGFR* loci

**Fig. 5:**
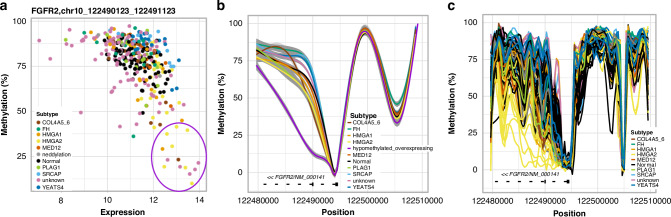
Methylation of *FGFR2* in ULs. **a** Total *FGFR2* expression level plotted against *FGFR2* promoter (1Kbp upstream from TSS) methylation level for each UL and myometrium sample with both RNA-seq and Nanopore data. Similar figures for each promoter region of alternative *FGFR2* transcripts can be found in the Supplementary material (Fig. S5). A group of ULs, mostly HMGA2/1 and unknown subtypes show promoter hypomethylation and overexpression of *FGFR2* (circled in purple). **b** Methylation levels per CpG island 15Kbp upstream and downstream from TSS in different UL subtypes and normal myometrium samples (only Nanopore batch 1 data). The samples circled with purple that were in batch 1 (the seven HMGA2 samples) in (**a**) are separated to their own subclass ‘hypomethylated_overexpressing’. **c** Methylation levels per CpG island 15Kbp upstream and downstream from TSS for each sample of Nanopore batch 1 data. HMGA2 ULs show clearly lower methylation of *FGFR2*.

We examined CpG methylation profiles 15Kbp upstream and downstream from *FGFR* TSSs (Fig. S4). HMGA2 ULs seemed to show slightly lower methylation levels around the second intron of *FGFR2* compared to other UL subtypes and normal myometrium samples (Fig. S4). We compared the average promoter methylation values to expression levels of *FGFR* genes and observed a group of ULs characterised by low methylation on promoters and high expression of *FGFR2* (Figs. 5a, S5). While these promoter regions belong to alternative transcript isoforms, in the canonical *FGFR2* transcript the hypomethylation pattern locates to the first intron and the second exon (Fig. 5b, c). These results suggest that hypomethylation of the promoter, first intron and second exon may be related to *FGFR2* overexpression in a subset of ULs. This group of ULs included mostly HMGA2/1 ULs, a few ULs without a known driver and one COL4A5/6 UL.

PLAG1 ULs showed a very clear *FGFR3* overexpression in RNA-seq compared to any other UL subtype or normal myometrium samples (log-fold-change 2.2–3.9, padj<1e-03) (Fig. 4e). In the promoter methylation analysis, the region of 1Kbp immediately upstream from *FGFR3* did not show hypomethylation. However, PLAG1 ULs showed slightly lower methylation levels about 3-8Kbp upstream from the *FGFR3* TSS compared to other UL subtypes (Fig. S4).

### *FGFRs* in UL predisposition

A GWAS comprising 58,390 UL cases and 493,433 female controls was inspected for inherited UL risk near the *FGFR* genes. The results revealed a genome-wide significant UL association locus 80Kbp upstream from *FGFR4*: The SNP rs353491 (hg38.chr5:177005946.C>G) was associated with a meta-analysis *P*-value of 4.2e-13 and the same risk allele (G) in all three cohorts: FINNGEN (odds ratio [OR] = 1.04, *P* = 4.8e-5), UKB (OR = 1.07, *P* = 7.0e-10) and BBJ (OR = 1.04, *P* = 4.8e-2). This region has been implicated also in previous UL-GWAS efforts, however, the target gene has remained unclear [41, 42]. Subsequent analysis of gene expression identified *FGFR4* as the most significant eQTL of the risk allele rs353491-G in both myometrium (log-fold-change = 0.32, *P* = 0.008) and tumour RNA-seq (log-fold-change = 0.27, *P* = 0.004). No genome-wide significant GWAS loci were found near *FGFR1*-*3*.

### Clinical information about the *FGFR* mutated ULs

The majority of the ULs with coding *FGFR* mutations, fusion gene, or *FGFR* gene gain and overexpression (10/12) were classified as conventional ULs in the histopathological evaluation (Table 1). The remaining two were evaluated as cellular and hypercellular ULs and one of them also atypical. The number of ULs varied from one to five in these patients. Compared to the other patients with somatic *FGFR* alterations, the patient with the germline *FGFR2* mutation (My6176) had the youngest diagnosis (33 y, mean in other patients 45.9) and hysterectomy age (35 y, mean in other patients 51.3). The tumour size was also the largest in this patient (∅ 17 cm), whereas the diameter of the other ULs with *FGFR* mutations ranged from 0.9 cm to 12 cm (Table 1).

**Table 1:** Clinical characteristics of the patients with coding *FGFR* mutations, fusion gene, or *FGFR* gene gain and overexpression.

UL sample ID	*FGFR* alteration	UL histological class	Tumour type	No of ULs/patient	UL subtype	UL subtype(s) of other myomas of the patient	UL size (Ø)	Size (Ø) of other ULs of the patient	Uterus weight	Age at diagnosis/hysterectomy
My6512m1	*FGFR1* p.Asn546Lys	Conventional	intramural	2	PLAG1	NA	4 cm	3 cm	95 g	52/60
My6715m1	*FGFR1* p.Asn546Lys	Conventional	intramural	2	HMGA1	MED12 (1)	10 cm	1.5 cm	440 g	61/65
My6292m1	*FGFR1* p.Asn546Asp	Conventional	subserous	3–5	HMGA2	MED12 (2)	5 cm	1.3 cm	218 g	42/45
My6649m1	*FGFR1* p.Lys656Glu	Conventional	NA	3	HMGA2	MED12 (2)	6 cm	1.8-3 cm	476 g	44/55
My6318m3	*FGFR2* p.Lys659Glu	Conventional	intramural	3	HMGA2	MED12 (2)	3 cm	6–11 cm	1326 g	47/48
My6068m1	*FGFR2* p.Met391Arg	Conventional	intramural	1	HMGA2	NA	12 cm	NA	860 g	35/39
My6176m1	*FGFR2* p.Pro363Ser (germline)	Conventional	intramural	1	HMGA1	NA	17 cm	NA	1340 g	33/35
My6006m1	*FGFR2-ERC1* fusion gene	Conventional	intramural	2	unknown	NA	0.9 cm	0.5 cm	144 g	47/52
My5007m1	*FGFR1* gain	Conventional	NA	2-3	HMGA1	unknown (2)	6 cm	4 cm	369 g	44/48
My5007m2	*FGFR1* gain	Conventional	NA	2-3	unknown	HMGA1 (1), unknown (1)	4 cm	6 cm	369 g	44/48
My6450m1	*FGFR1* gain	Hypercellular, atypical	intramural	2	unknown	unknown (1)	5 cm	3.5 cm	292 g	47/52
My6467m2	*FGFR1* gain	Cellular	NA	5	HMGA1	MED12 (4)	2 cm	2.5–6 cm	358 g	42/52

## Discussion

In this study, we have shown that *FGFR* genes are activated in a subset of ULs via multiple mechanisms. We encountered hotspot mutations in *FGFR1/2*, other coding mutations in *FGFR2*, copy number gains leading to overexpression of *FGFR1* and an *FGFR2* fusion gene. In addition to genetic alterations, our results indicate that hypomethylation of regulatory regions at *FGFR2* and *FGFR3* and consequent overexpression may contribute to UL genesis.

Altogether, five out of the eight *FGFR1/2* point mutations detected in our UL collection, occurred in the highly specific mutation hotspots that are established drivers of tumourigenesis. All the point mutations in *FGFR1* occurred in hotspot amino acids (p.Asn546Lys, p.Asn546Asp, p.Lys656Glu) that have previously been reported in many cancer types, including glioma and breast carcinoma [15]. *FGFR2* p.Lys659Glu, which is homologous to aforementioned *FGFR1* p.Lys656Glu, has earlier been seen in breast and endometrial carcinomas [15].

The AlphaMissense pathogenicity prediction for *FGFR2* p.Met391Arg is as high as for the hotspot mutations, suggesting that it probably is pathogenic (Table S3). The predicted pathogenicity value is much lower for the only germline mutation found in this study, *FGFR2* p.Pro363Ser. However, this mutation is not a known variant in the gnomAD database [28]. The youngest age at diagnosis and hysterectomy of this patient as well as the largest tumour size compared to somatically mutated *FGFR* cases indicate that this germline change may predispose to UL. Our GWAS results support the notion that inherited *FGFR*-related variants play a role in UL predisposition, as we found a genome-wide significant locus 80Kbp upstream from *FGFR4* associated with the UL phenotype in the FINNGEN, BBJ and UKB cohorts. The most significant eQTL for the risk allele was *FGFR4*; the risk allele carriers showed increased *FGFR4* expression in both myometrium (*P* = 0.008) and ULs (*P* = 0.004).

Here, we also report an in-frame *FGFR2-ERC1* fusion gene in one UL. In this alteration all the functional domains of *FGFR2* were preserved. The regions encoding functionally important coiled-coil domains of *ERC1* were preserved for the most parts. A similar *FGFR2-ERC1* fusion gene has previously been reported in lung adenocarcinoma [43]. *ERC1* has also been reported as a known fusion partner gene for other transmembrane tyrosine kinase receptors, *RET* and *ALK*. *ERC1-RET* fusions have been reported in pancreatic ductal adenocarcinoma, papillary thyroid carcinoma and lung cancer [44–46]. In turn, *ERC1*-*ALK* fusions have been seen in sarcomas and non–small cell lung carcinoma [47, 48]. *ERC1* encodes for ELKS/RAB6-Interacting/CAST Family Member 1, which functions as a scaffold on the active zone on presynaptic plasma membrane affecting membrane trafficking, as a regulator of focal adhesion disassembly and in the regulation of insulin secretion [49–51].

We found altogether four ULs, of which two clonally related, with gains of chromosome 8 associated with *FGFR1* overexpression. *FGFR1* gains and amplifications are the most common *FGFR* alterations in cancer [16]. Chromosomal rearrangements are known to activate *FGFR* genes in cancer [14]. In our dataset, we found one *FGFR4* overexpressing UL with multiple SVs in chromosome 5 with the closest breakpoints from *FGFR4* about 491Kbp upstream that may explain the slight *FGFR4* overexpression.

Investigation of *FGFR* methylation levels suggests that *FGFR2* and *FGFR3* hypomethylation may lead to overexpression of these genes in a subset of ULs. *FGFR2* hypomethylation was seen in the first intron of the canonical transcript that is the promoter region of a few alternative transcripts (Fig. S5). The *FGFR2* overexpression was not limited to those transcripts with hypomethylated promoter regions but also transcripts with hypomethylated intron 1 were overexpressed. Indeed, it has been shown that similarly to promoter hypomethylation also the hypomethylation of intron 1 correlates with higher gene expression levels, providing further evidence on the correlation between *FGFR2* intronic hypomethylation and increased gene expression [52]. *FGFR3* expression was significantly higher in PLAG1 ULs compared to other UL subtypes and normal myometrium. Methylation profiles 15Kbp upstream and downstream of *FGFR3* TSS suggest that hypomethylation of the region around 3-8Kbp upstream from *FGFR3* may explain the *FGFR3* overexpression in at least some of the PLAG1 ULs (Fig. S4).

Interestingly, the great majority (9/12) of the coding *FGFR* alterations (point mutations in coding regions, the *FGFR2-ERC1* fusion, and *FGFR1* gain with overexpression) were seen in ULs of HMGA2, HMGA1 and PLAG1 subtypes, suggesting that *FGFR* alterations are more typical for these subtypes. These three UL subtypes are known to be molecularly similar to each other, probably driving tumorigenesis through *PLAG1* overexpression [11]. Indeed, statistical analysis revealed a significant enrichment of coding *FGFR* alterations in HMGA2/1 and PLAG1 ULs (OR = 10.2, *P* = 1.9e-04, Fisher’s exact test). This also explains why we only found one *FGFR1/2* mutation in the extensive additional Sanger screening effort, as a large proportion of HMGA1/2 and PLAG1 tumours had been included in the discovery set of RNA-sequenced samples (*MED12* mutations explain for the large majority of all UL cases, and thus, up to 75.3% [2,016/2,677] of ULs in our unselective Finland Myoma Study collection belong to MED12 subtype) [5, 9]. The prevalence of activating genetic *FGFR* alterations was 0.45% (12/2,677) in unselected ULs and the estimated prevalence in HMGA2/1 and PLAG1 ULs is 3.0% (9/302). In addition, hypomethylation with overexpression of *FGFR2* was seen especially in HMGA2 ULs and of *FGFR3* in PLAG1 ULs.

It is under debate whether ULs can serve as a precursor lesion for uterine leiomyosarcomas (ULMS). Although most ULMS do not share common genetic drivers with ULs, *MED12* hotspot mutations, HMGA2 overexpression and *FH* inactivation have been shown to occur in some ULMS cases, suggesting that leiomyosarcomas may originate from Uls [53]. As *FGFR* activating alterations are typical in many different cancer types, the question arises if *FGFR* mutated ULs have a higher potential to develop malignancy. To our knowledge, *FGFR* hotspot mutations have not been reported in ULMS but *FGFR1* duplications, *FGFR3* amplifications and deletions and *FGFR3/4* overexpression have been identified in these tumours [54, 55]. The genetic landscape of ULMS is not very widely studied, and more research is needed to tackle this question.

Our findings establish the role of FGFR signalling in UL genesis, as previously suggested by others [17–19]. Currently, all the curative treatment options for ULs are invasive, and ULs are treated as one entity although differences in drug responses have been shown between UL subtypes [3, 56]. Our results raise the question if FGFR inhibitors could be feasible for treating *FGFR* mutated ULs in the future, if uninvasive molecular classification of these tumours by cell-free DNA or similar approaches becomes feasible [57]. Three FGFR inhibitors are currently approved for clinical use by FDA: erdafitinib for metastatic or locally advanced urothelial carcinoma with *FGFR3* alterations; pemigatinib and futibatinib for metastatic or locally advanced, unresectable and earlier treated cholangiocarcinoma with *FGFR2* fusion or rearrangement, and pemigatinib also for relapsed or refractory myeloid or lymphoid neoplasms with *FGFR1* rearrangements [58, 59]. FGFR inhibitors are known to have adverse effects, such as hyperphosphatemia, ocular symptoms, and alopecia, and for management of a benign disease extensive side effects cannot be accepted [60]. However, multiple novel FGFR inhibitors are being developed for various tumour types with *FGFR* alterations [58]. New FGFR inhibitors with milder adverse effects could potentially be applicable for *FGFR* driven ULs in the future.

## Supplementary information

Supplemental material

Supplemental information

Supplemental information

Supplemental information

## Data Availability

The biobank datasets used during this study are available at UK Biobank (https://www.ukbiobank.ac.uk/), FINNGEN (https://finngen.fi/) and Biobank Japan (http://jenger.riken.jp/en/). Genetic data supporting the findings presented in this manuscript.

## References

[1] Baird DD, Dunson DB, Hill MC, Cousins D, Schectman JM. High cumulative incidence of uterine leiomyoma in black and white women: ultrasound evidence. Am J Obstet Gynecol. 2003;188:100–7. 12548202 10.1067/mob.2003.99

[2] Stewart EA, Cookson CL, Gandolfo RA, Schulze-Rath R. Epidemiology of uterine fibroids: a systematic review. BJOG. 2017;124:1501–12. 28296146 10.1111/1471-0528.14640

[3] Al-Hendy A, Myers ER, Stewart E. Uterine fibroids: burden and unmet medical need. Semin Reprod Med. 2017;35:473–80. 29100234 10.1055/s-0037-1607264 PMC6193285

[4] Cardozo ER, Clark AD, Banks NK, Henne MB, Stegmann BJ, Segars JH. The estimated annual cost of uterine leiomyomata in the United States. Am J Obstet Gynecol. 2012;206:211.e1–9. 22244472 10.1016/j.ajog.2011.12.002 PMC3292655

[5] Mäkinen N, Mehine M, Tolvanen J, Kaasinen E, Li Y, Lehtonen HJ, et al. MED12, the mediator complex subunit 12 gene, is mutated at high frequency in uterine leiomyomas. Science. 2011;334:252–5. 21868628 10.1126/science.1208930

[6] Mehine M, Kaasinen E, Heinonen HR, Mäkinen N, Kämpjärvi K, Sarvilinna N, et al. Integrated data analysis reveals uterine leiomyoma subtypes with distinct driver pathways and biomarkers. Proc Natl Acad Sci USA. 2016;113:1315–20. 26787895 10.1073/pnas.1518752113 PMC4747776

[7] Tomlinson IPM, Alam NA, Rowan AJ, Barclay E, Jaeger EEM, Kelsell D, et al. Germline mutations in FH predispose to dominantly inherited uterine fibroids, skin leiomyomata and papillary renal cell cancer. Nat Genet. 2002;30:406–10. 11865300 10.1038/ng849

[8] Lehtonen R, Kiuru M, Vanharanta S, Sjöberg J, Aaltonen L-M, Aittomäki K, et al. Biallelic inactivation of fumarate hydratase (FH) occurs in nonsyndromic uterine leiomyomas but is rare in other tumors. Am J Pathol. 2004;164:17–22. 14695314 10.1016/S0002-9440(10)63091-X PMC1602244

[9] Berta DG, Kuisma H, Välimäki N, Räisänen M, Jäntti M, Pasanen A, et al. Deficient H2A.Z deposition is associated with genesis of uterine leiomyoma. Nature. 2021;596:398–403. 10.1038/s41586-021-03747-1 34349258 10.1038/s41586-021-03747-1

[10] Mehine M, Ahvenainen T, Khamaiseh S, Härkönen J, Reinikka S, Heikkinen T, et al. A novel uterine leiomyoma subtype exhibits NRF2 activation and mutations in genes associated with neddylation of the Cullin 3-RING E3 ligase. Oncogenesis. 2022;11:52. 36068196 10.1038/s41389-022-00425-3 PMC9448808

[11] Jokinen V, Mehine M, Reinikka S, Khamaiseh S, Ahvenainen T, Äyräväinen A, et al. 3’RNA and whole-genome sequencing of archival uterine leiomyomas reveal a tumor subtype with chromosomal rearrangements affecting either HMGA2, HMGA1, or PLAG1. Genes Chromosomes Cancer. 2023;62:27–38. 35822448 10.1002/gcc.23088 PMC9804854

[12] Mehine M, Mäkinen N, Heinonen HR, Aaltonen LA, Vahteristo P. Genomics of uterine leiomyomas: Insights from high-throughput sequencing. Fertil Steril. 2014;102:621–9. 25106763 10.1016/j.fertnstert.2014.06.050

[13] Katoh M, Nakagama H. FGF receptors: cancer biology and therapeutics. Med Res Rev. 2014;34:280–300. 23696246 10.1002/med.21288

[14] Helsten T, Elkin S, Arthur E, Tomson BN, Carter J, Kurzrock R. The FGFR landscape in cancer: analysis of 4853 tumors by next-generation sequencing. Clin Cancer Res. 2016;22:259–67. 26373574 10.1158/1078-0432.CCR-14-3212

[15] Tate JG, Bamford S, Jubb HC, Sondka Z, Beare DM, Bindal N, et al. COSMIC: the catalogue of somatic mutations in cancer. Nucleic Acids Res. 2019;47:D941–D947. 30371878 10.1093/nar/gky1015 PMC6323903

[16] Krook MA, Reeser JW, Ernst G, Barker H, Wilberding M, Li G, et al. Fibroblast growth factor receptors in cancer: genetic alterations, diagnostics, therapeutic targets and mechanisms of resistance. Br J Cancer. 2021;124:880–92. 33268819 10.1038/s41416-020-01157-0 PMC7921129

[17] Wu X, Blanck A, Olovsson M, Möller B, Lindblom B. Expression of basic fibroblast growth factor (bFGF), FGF receptor 1 and FGF receptor 2 in uterine leiomyomas and myometrium during the menstrual cycle, after menopause and GnRHa treatment. Acta Obstet Gynecol Scand. 2001;80:497–504. 11380284

[18] Anania CA, Stewart EA, Quade BJ, Hill JA, Nowak RA. Expression of the fibroblast growth factor receptor in women with leiomyomas and abnormal uterine bleeding. Mol Hum Reprod. 1997;3:685–91. 9294852 10.1093/molehr/3.8.685

[19] Cirilo PD, Marchi FA, Barros Filho Mde C, Rocha RM, Domingues MA, Jurisica I, et al. An integrative genomic and transcriptomic analysis reveals potential targets associated with cell proliferation in uterine leiomyomas. PLoS ONE. 2013;8:e57901. 23483937 10.1371/journal.pone.0057901 PMC3587425

[20] Mehine M, Kaasinen E, Mäkinen N, Katainen R, Kämpjärvi K, Pitkänen E, et al. Characterization of uterine leiomyomas by whole-genome sequencing. N Engl J Med. 2013;369:43–53. 23738515 10.1056/NEJMoa1302736

[21] Mäkinen N, Vahteristo P, Bützow R, Sjöberg J, Aaltonen LA. Exomic landscape of MED12 mutation-negative and -positive uterine leiomyomas. Int J Cancer. 2014;134:1008–12. 23913526 10.1002/ijc.28410

[22] Love MI, Huber W, Anders S. Moderated estimation of fold change and dispersion for RNA-seq data with DESeq2. Genome Biol. 2014;15:550. 25516281 10.1186/s13059-014-0550-8 PMC4302049

[23] Ritchie ME, Phipson B, Wu D, Hu Y, Law CW, Shi W, et al. limma powers differential expression analyses for RNA-sequencing and microarray studies. Nucleic Acids Res. 2015;43:e47. 25605792 10.1093/nar/gkv007 PMC4402510

[24] Dobin A, Davis CA, Schlesinger F, Drenkow J, Zaleski C, Jha S, et al. STAR: ultrafast universal RNA-seq aligner. Bioinformatics. 2013;29:15–21. 23104886 10.1093/bioinformatics/bts635 PMC3530905

[25] Van der Auwera GA, O’Connor BD. Genomics in the cloud: using docker, GATK, and WDL in Terra, 1st ed. (O’Reilly Media: Sebastopol, CA, USA, 2020).

[26] Morales J, Pujar S, Loveland JE, Astashyn A, Bennett R, Berry A, et al. A joint NCBI and EMBL-EBI transcript set for clinical genomics and research. Nature. 2022;604:310–5. 35388217 10.1038/s41586-022-04558-8 PMC9007741

[27] Katainen R, Donner I, Cajuso T, Kaasinen E, Palin K, Mäkinen V, et al. Discovery of potential causative mutations in human coding and noncoding genome with the interactive software BasePlayer. Nat Protoc. 2018;13:2580–2600. 30323186 10.1038/s41596-018-0052-3

[28] Chen S, Francioli LC, Goodrich JK, Collins RL, Kanai M, Wang Q, et al. A genomic mutational constraint map using variation in 76,156 human genomes. Nature. 2024;625:92–100. 38057664 10.1038/s41586-023-06045-0 PMC11629659

[29] Wang K, Li M, Hakonarson H. ANNOVAR: functional annotation of genetic variants from high-throughput sequencing data. Nucleic Acids Res. 2010;38:e164. 20601685 10.1093/nar/gkq603 PMC2938201

[30] Paysan-Lafosse T, Blum M, Chuguransky S, Grego T, Pinto BL, Salazar GA, et al. InterPro in 2022. Nucleic Acids Res. 2023;51:D418–D427. 36350672 10.1093/nar/gkac993 PMC9825450

[31] Nicorici D, Şatalan M, Edgren H, Kangaspeska S, Murumägi A, Kallioniemi O, et al. FusionCatcher—a tool for finding somatic fusion genes in paired-end RNA-sequencing data. bioRxiv. 2014; 011650.

[32] Karolchik D, Hinrichs AS, Furey TS, Roskin KM, Sugnet CW, Haussler D, et al. The UCSC Table Browser data retrieval tool. Nucleic Acids Res. 2004;32:D493–6. 10.1093/nar/gkh103 PMC308837 14681465

[33] Wickham H. ggplot2: elegant graphics for data analysis. Springer Science & Business Media; 2009 https://play.google.com/store/books/details?id=bes-AAAAQBAJ.

[34] Danecek P, Bonfield JK, Liddle J, Marshall J, Ohan V, Pollard MO, et al. Twelve years of SAMtools and BCFtools. Gigascience. 2021;10:giab008. 10.1093/gigascience/giab008 33590861 10.1093/gigascience/giab008 PMC7931819

[35] Minton K. Predicting variant pathogenicity with AlphaMissense. Nat Rev Genet. 2023;24:804. 37821682 10.1038/s41576-023-00668-9

[36] Jiang T, Liu Y, Jiang Y, Li J, Gao Y, Cui Z, et al. Long-read-based human genomic structural variation detection with cuteSV. Genome Biol. 2020;21:189. 32746918 10.1186/s13059-020-02107-y PMC7477834

[37] Jiang T, Cao S, Liu Y, Liu S, Liu B, Wang G et al. Regenotyping structural variants through an accurate force-calling method. bioRxiv. 2023; 2022.08.29.505534.

[38] Thorvaldsdóttir H, Robinson JT, Mesirov JP. Integrative Genomics Viewer (IGV): high-performance genomics data visualization and exploration. Brief Bioinform. 2013;14:178–92. 22517427 10.1093/bib/bbs017 PMC3603213

[39] Van Loo P, Nordgard SH, Lingjærde OC, Russnes HG, Rye IH, Sun W, et al. Allele-specific copy number analysis of tumors. Proc Natl Acad Sci USA. 2010;107:16910–5. 20837533 10.1073/pnas.1009843107 PMC2947907

[40] Kurki MI, Karjalainen J, Palta P, Sipilä TP, Kristiansson K, Donner KM, et al. FinnGen provides genetic insights from a well-phenotyped isolated population. Nature. 2023;613:508–18. 36653562 10.1038/s41586-022-05473-8 PMC9849126

[41] Välimäki N, Kuisma H, Pasanen A, Heikinheimo O, Sjöberg J, Bützow R, et al. Genetic predisposition to uterine leiomyoma is determined by loci for genitourinary development and genome stability. Elife. 2018;7:e37110 10.7554/eLife.37110 30226466 10.7554/eLife.37110 PMC6203434

[42] Rafnar T, Gunnarsson B, Stefansson OA, Sulem P, Ingason A, Frigge ML, et al. Variants associating with uterine leiomyoma highlight genetic background shared by various cancers and hormone-related traits. Nat Commun. 2018;9:3636. 30194396 10.1038/s41467-018-05428-6 PMC6128903

[43] Hong C, Wei J, Zhou T, Wang X, Cai J. FGFR2-ERC1: a subtype of FGFR2 oncogenic fusion variant in lung adenocarcinoma and the response to anlotinib. Onco Targets Ther. 2022;15:651–7. 35712652 10.2147/OTT.S364566 PMC9196998

[44] Ma J, Wang B, Meng E, Meng X. Case report: identification of ERC1-RET fusion in a patient with pancreatic ductal adenocarcinoma. Gland Surg. 2021;10:2874–9. 34733735 10.21037/gs-21-469 PMC8514310

[45] Liu Y, Wu S, Zhou L, Guo Y, Zeng X. Pitfalls in RET fusion detection using break-apart FISH probes in papillary thyroid carcinoma. J Clin Endocrinol Metab. 2021;106:1129–38. 33382428 10.1210/clinem/dgaa913

[46] Shi M, Wang W, Zhang J, Li B, Lv D, Wang D, et al. Identification of RET fusions in a Chinese multicancer retrospective analysis by next-generation sequencing. Cancer Sci. 2022;113:308–18. 34710947 10.1111/cas.15181 PMC8748217

[47] Chen T, Wang Y, Goetz L, Corey Z, Dougher MC, Smith JD, et al. Novel fusion sarcomas including targetable NTRK and ALK. Ann Diagn Pathol. 2021;54:151800. 34464935 10.1016/j.anndiagpath.2021.151800

[48] Couëtoux du Tertre M, Marques M, Tremblay L, Bouchard N, Diaconescu R, Blais N, et al. Analysis of the genomic landscape in ALK+ NSCLC patients identifies novel aberrations associated with clinical outcomes. Mol Cancer Ther. 2019;18:1628–36. 31243098 10.1158/1535-7163.MCT-19-0105

[49] Held RG, Kaeser PS. ELKS active zone proteins as multitasking scaffolds for secretion. Open Biol. 2018;8:170258 10.1098/rsob.170258 29491150 10.1098/rsob.170258 PMC5830537

[50] Astro V, Chiaretti S, Magistrati E, Fivaz M, de Curtis I. Liprin-α1, ERC1 and LL5 define polarized and dynamic structures that are implicated in cell migration. J Cell Sci. 2014;127:3862–76. 24982445 10.1242/jcs.155663

[51] Ohara-Imaizumi M, Aoyagi K, Ohtsuka T. Role of the active zone protein, ELKS, in insulin secretion from pancreatic β-cells. Mol Metab. 2019;27S:S81–S91. 31500835 10.1016/j.molmet.2019.06.017 PMC6768504

[52] Anastasiadi D, Esteve-Codina A, Piferrer F. Consistent inverse correlation between DNA methylation of the first intron and gene expression across tissues and species. Epigenetics Chromatin. 2018;11:37. 29958539 10.1186/s13072-018-0205-1 PMC6025724

[53] Mäkinen N, Kämpjärvi K, Frizzell N, Bützow R, Vahteristo P. Characterization of MED12, HMGA2, and FH alterations reveals molecular variability in uterine smooth muscle tumors. Mol Cancer. 2017;16:1–8. 28592321 10.1186/s12943-017-0672-1 PMC5463371

[54] Mas A, Alonso R, Garrido-Gómez T, Escorcia P, Montero B, Jiménez-Almazán J, et al. The differential diagnoses of uterine leiomyomas and leiomyosarcomas using DNA and RNA sequencing. Am J Obstet Gynecol. 2019;221:320.e1–320.e23. 31121144 10.1016/j.ajog.2019.05.018

[55] Selenica P, Conlon N, Gonzalez C, Frosina D, Jungbluth AA, Beets-Tan RGH, et al. Genomic profiling aids classification of diagnostically challenging uterine mesenchymal tumors with myomelanocytic differentiation. Am J Surg Pathol. 2021;45:77–92. 32889887 10.1097/PAS.0000000000001572 PMC8276853

[56] Kolterud Å, Välimäki N, Kuisma H, Patomo J, Ilves ST, Mäkinen N, et al. Molecular subclass of uterine fibroids predicts tumor shrinkage in response to ulipristal acetate. Hum Mol Genet. 2022. 10.1093/hmg/ddac217. 10.1093/hmg/ddac217 PMC10026225 36048862

[57] Tamehisa T, Sato S, Sakai T, Maekawa R, Tanabe M, Ito K, et al. Establishment of noninvasive prediction models for the diagnosis of uterine leiomyoma subtypes. Obstet Gynecol. 2024;143:358. 38061038 10.1097/AOG.0000000000005475

[58] Katoh M, Loriot Y, Brandi G, Tavolari S, Wainberg ZA, Katoh M. FGFR-targeted therapeutics: clinical activity, mechanisms of resistance and new directions. Nat Rev Clin Oncol. 2024;21:312–29. 38424198 10.1038/s41571-024-00869-z

[59] Center for Drug Evaluation, Research. FDA approves pemigatinib for relapsed or refractory myeloid/lymphoid neoplasms with FGFR1 rearrangement. U.S. Food and Drug Administration. 2022. https://www.fda.gov/drugs/resources-information-approved-drugs/fda-approves-pemigatinib-relapsed-or-refractory-myeloidlymphoid-neoplasms-fgfr1-rearrangement. Accessed 14 Jun 2024.

[60] Subbiah V, Verstovsek S. Clinical development and management of adverse events associated with FGFR inhibitors. Cell Rep Med. 2023;4:101204. 37757826 10.1016/j.xcrm.2023.101204 PMC10591034

