# Supplementary material for: Activation of FGFR genes by genetic and epigenetic alterations in uterine leiomyomas
Source: BJC Rep. 2025 Feb 27;3:9. doi: 10.1038/s44276-025-00127-4 (PMC11868550; doi:10.1038/s44276-025-00127-4)
Supplement: Supplementary file 1 — Supplemental material [file 44276_2025_127_MOESM1_ESM.docx]

**Supplemental material**

**Supplemental material and methods**

**UL subtype detection**

The Finland Myoma Study sample set was screened for known driver alterations. All the UL samples were screened for *MED12* hotspot mutations by Sanger sequencing, and in all mutation-free samples HMGA2 expression was assessed using immunohistochemistry (IHC) or qPCR as earlier described.[^1^](https://paperpile.com/c/xAjzEJ/ry5nT) ULs without *MED12* or HMGA2 alterations entered H2A.Z IHC and ULs with reduced H2A.Z staining were sequenced for SRCAP complex gene (*ACTL6A, DMAP1, VPS72, YEATS4,* and *ZNHIT1)* mutations. ULs that showed chr1q deletions on SNP array data or were AKR1B10 IHC positive were screened for *FH* mutations.[^2^](https://paperpile.com/c/xAjzEJ/UdYXe) If *FH* mutation was not detected tumours were further screened for Cullin 3-RING E3 ligase neddylation defects (*CUL3, NAE1, NEDD8,* and *UBE2M* mutations).[^3^](https://paperpile.com/c/xAjzEJ/HQ9KF)

For the ULs with available Nanopore or whole-genome sequencing[^4^](https://paperpile.com/c/xAjzEJ/pwUUV) data (Table S1.), HMGA1 and PLAG1 subtypes were defined based on structural variants (SV) with a breakpoint maximum 500Kbp from these two genes. If available, RNA-seq data was used to assign HMGA1 and PLAG1 status in a subset of ULs without known driver alteration and Nanopore data. The sample was defined as HMGA1 UL if the *PLAG1* and *HMGA1* expression levels exceeded the following thresholds calculated based on the ULs with a known *HMGA1* SV: mean - standard deviation (SD) for *HMGA1* and mean - 2*SD for *PLAG1*. Similarly, the sample was defined as PLAG1 UL if *PLAG1* expression was higher than the mean - 2*SD expression calculated in ULs with *PLAG1* SV but no *HMGA1* overexpression was seen (Figure S1).

ULs were defined as COL4A5-COL4A6 subtype if UL had a manually curated deletion in COL4A5-6 locus on SNP chip data based on decreased logR ratio compared to the sample mean. ULs showing an SV with a breakpoint within 500kB downstream from *IRS4* on Nanopore data were also defined as COL4A5-6 ULs. In addition, if UL showed *IRS4* overexpression and downregulated COL4A5/6 expression, UL was assigned to the COL4A5-6 subtype.

**Long-read sequencing**

The Nanopore long-read sequencing was performed for 283 ULs and 106 normal myometrium samples. For the sequencing library preparation, the Ligation Sequencing Kit LSK-109, LSK-110 or LSK-114 (Oxford Nanopore Technologies) was used according to the manufacturer’s Genomic DNA by Ligation protocol. The sequencing was performed on the PromethION platform with FLO-PRO001, FLO-PRO002, FLO-PRO114M and base and methylation calling with guppy/megalodon 2.4.2 dna_r9.4.1_450bps_hac_prom model (Batch 1, n=217) or with live basecalling model dna_r10.4.1_e8.2_400bps_hac (Batch 2, n=172). Reads were aligned against the T2T-CHM13v2.0 reference genome using minimap2 v.2.16.[^5^](https://paperpile.com/c/xAjzEJ/MwNS) Phasing to haplotypes and SNV calling was done with Longshot v.0.4.3.[^6^](https://paperpile.com/c/xAjzEJ/MXCa) For the data quality evaluation, we used NanoStat v.1.1.2 and NanoPlot v.1.20.0.[^7,8^](https://paperpile.com/c/xAjzEJ/CQSF+wNg7)

**Table S1. Uterine leiomyoma (UL) sample set used in this study and data types available for each sample.** The sample set of this study consisted of 2,677 ULs and corresponding myometrium samples from 863 patients. In the *RNAseq_data* column “low_coverage” means that the minimum read depth was less than 15 for at least one of the TOP3 *FGFR1* hotspots or TOP4 *FGFR2* hotspots according to the COSMIC database and thus, the sample was screened for *FGFR1/2* mutations by another method. The table given as a separate file.

**Table S2. Mutation calls from the RNA-sequencing data (n=410) of the genes with at least four different mutations in four different UL samples.** *MED12*, *FH,* and SRCAP complex genes (*DMAP1*, *ACTL6A*, and *YEATS4*) are previously reported well-known driver genes in ULs. The table given as a separate file.

**Table S3. *FGFR* gene mutations found in the RNA-sequencing data set of 410 ULs and Sanger screening of *FGFR1/2* mutation hotspots.**

| **Sample​** | **Subtype​** | **Gene​** |  | **Coding**  **effect​** | **Protein effect​** | **Mutation type​** | **Population Allele Frequency (gnomAD)​** | **Alpha Missense prediction​** | **Discovery Method​** | **Validation method​** |
| --- | --- | --- | --- | --- | --- | --- | --- | --- | --- | --- |
| My6512m1​ | PLAG1​ | *FGFR1*​ |  | c.1638C>G​ | p.Asn546Lys​ | somatic​ | 0​ | 0.99​ | RNA-seq​ | Sanger, Nanopore​ |
| My6715m1​ | HMGA1​ | *FGFR1*​ |  | c.1638C>A​ | p.Asn546Lys​ | somatic​ | 0​ | 0.99​ | RNA-seq​ | Sanger, Nanopore​ |
| My6292m1​ | HMGA2​ | *FGFR1*​ |  | c.1636A>G​ | p.Asn546Asp​ | somatic​ | 0​ | 0.89​ | RNA-seq​ | Sanger, WGS​ |
| My6649m1​ | HMGA2​ | *FGFR1*​ |  | c.1966A>G​ | p.Lys656Glu​ | somatic​ | 0​ | 1​ | Sanger ​ | Sanger​ |
| My6318m3​ | HMGA2​ | *FGFR2*​ |  | c.1975A>G​ | p.Lys659Glu​ | somatic​ | 0​ | 1​ | RNA-seq | Sanger, WGS​ |
| My6057m4​ | MED12, COL4A5_6​ | *FGFR2*​ |  | c.1288-17T>G​ | NA​ | somatic​ | 0​ | NA​ | RNA-seq​ | Sanger​ |
| My6068m1​ | HMGA2​ | *FGFR2*​ |  | c.1172T>G​ | p.Met391Arg​ | somatic​ | 0​ | 0.99​ | RNA-seq​ | Sanger, WGS​ |
| My6176m1​ | HMGA1​ | *FGFR2*​ |  | c.1087C>T​ | p.Pro363Ser​ | germline​ | 0​ | 0.1​ | RNA-seq​ | Sanger​ |

**Table S4. Structural variants (SV) of *FGFR1-4* from the Nanopore data of ULs (n=283).** The SVs with a breakpoint within 1Mbp from *FGFR1-4* are included to the list. The only intragenic breakpoints were seen in UL My6006m1 with the *FGFR2-ERC1* fusion. The table given as a separate file.

**Table S5. Copy number gains overlapping *FGFR* genes found in SNP array data of 2,030 UL samples.** The coordinates of gained regions are given according to the GRCh38 reference genome. If the gain was found by both AI and somatic copy number variation analysis, the combined region is reported. The samples marked with an asterisk (*) are clonally related.

| **Sample**​ | **UL subtype**​ | **Chr**​ | **Start**​ | **End**​ | **Gene**​ | **Expression data**​ | **Target *FGFR* gene overexpression**​ |
| --- | --- | --- | --- | --- | --- | --- | --- |
| My6203m1​ | neddylation​ | 8​ | 213226​ | 42946512​ | *FGFR1*​ | RNA-seq​ | no​ |
| My6106m1​ | neddylation​ | 8​ | 213226​ | 145050580​ | *FGFR1*​ | RNA-seq​ | no​ |
| My6467m2​ | HMGA1​ | 8​ | 213226​ | 116381947​ | *FGFR1*​ | RNA-seq​ | yes​ |
| My6298m1​ | HMGA2​ | 8​ | 213226​ | 145050580​ | *FGFR1*​ | qPCR​ | no​ |
| My5007m1*​ | HMGA1​ | 8​ | 226818​ | 121373205​ | *FGFR1*​ | RNA-seq​ | yes​ |
| My6450m1​ | unknown​ | 8​ | 2145257​ | 140335539​ | *FGFR1*​ | RNA-seq​ | yes​ |
| My6377m9​ | MED12​ | 8​ | 30372803​ | 97764651​ | *FGFR1*​ | qPCR​ | no​ |
| My5007m2​* | unknown​ | 8​ | 31773467​ | 97870524​ | *FGFR1*​ | qPCR​ | yes​ |
| My6523m8​ | SRCAP​ | 10​ | 67994​ | 133620799​ | *FGFR2*​ | RNA-seq​ | no​ |
| My6106m1​ | neddylation​ | 4​ | 71674​ | 27207937​ | *FGFR3*​ | RNA-seq​ | no​ |
| My6450m1​ | unknown​ | 4​ | 85531​ | 189824738​ | *FGFR3*​ | RNA-seq​ | no​ |
| My6298m1​ | HMGA2​ | 4​ | 71674​ | 189959254​ | *FGFR3*​ | NA​ | NA​ |
| My6218m5​ | MED12​ | 4​ | 71674​ | 189959254​ | *FGFR3*​ | NA​ | NA​ |
| My6181m1​ | MED12​ | 4​ | 71674​ | 189959254​ | *FGFR3*​ | NA​ | NA​ |
| My6484m1​ | MED12​ | 4​ | 71674​ | 189959254​ | *FGFR3*​ | NA​ | NA​ |
| My6203m1​ | neddylation​ | 4​ | 704503​ | 22162694​ | *FGFR3*​ | RNA-seq​ | no​ |
| My6350m1​ | HMGA1​ | 4​ | 71674​ | 2813529​ | *FGFR3*​ | RNA-seq​ | no​ |
| My6106m1​ | neddylation​ | 5​ | 48440​ | 181263936​ | *FGFR4*​ | RNA-seq​ | no​ |
| My6298m1​ | HMGA2​ | 5​ | 38141​ | 181263936​ | *FGFR4*​ | NA​ | NA​ |
| My6349m1​ | MED12​ | 5​ | 38141​ | 181263936​ | *FGFR4*​ | NA​ | NA​ |
| My6203m1​ | neddylation​ | 5​ | 45477906​ | 181263936​ | *FGFR4​* | RNA-seq​ | no​ |
| My6450m1​ | unknown​ | 5​ | 131498253​ | 181199927​ | *FGFR4*​ | RNA-seq​ | no​ |


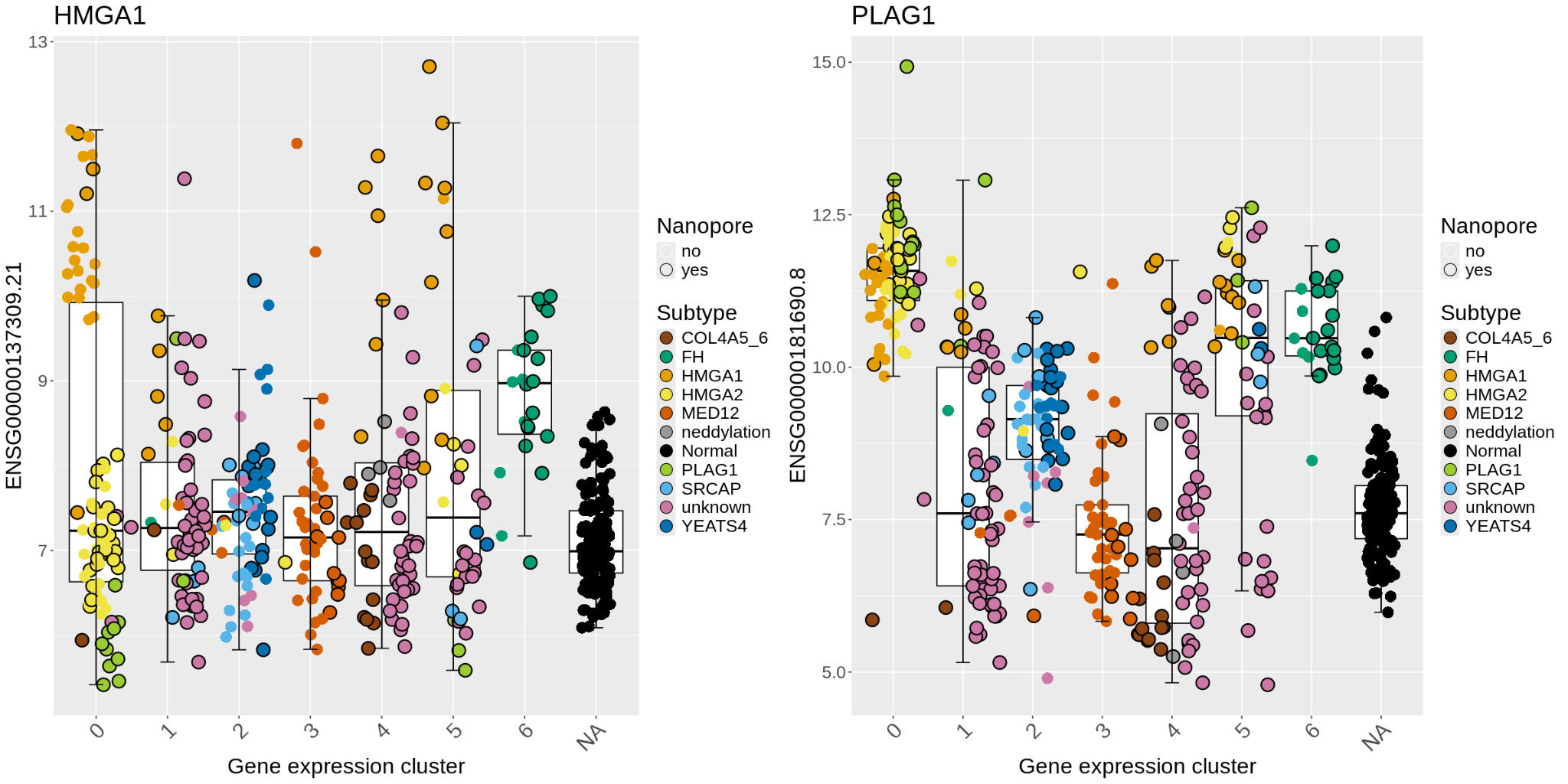


**Figure S1. *HMGA1* and *PLAG1* expression levels in UL and myometrium samples in the RNA-sequencing data.** Samples are coloured by the tumour subtype and the tumours are grouped by unsupervised hierarchical clustering based on global gene expression. ULs with *HMGA1* or *PLAG1* SVs detected in Nanopore long-read sequencing were assigned to HMGA1 and PLAG1 subtypes. For samples with no Nanopore data available and no other known driver alteration, HMGA1 and PLAG1 statuses were determined based on expression data: UL samples with *HMGA1* and *PLAG1* overexpression were added to the HMGA1 subtype, and ULs showing *PLAG1* overexpression but no *HMGA1* overexpression were assigned to the PLAG1 subtype. All except one of these HMGA1/PLAG1 ULs defined by gene expression levels were in cluster 0 that also includes majority of ULs of HMGA2 subtype that is known to be molecularly similar to HMGA1 and PLAG1 ULs.[^9^](https://paperpile.com/c/xAjzEJ/2YQr)


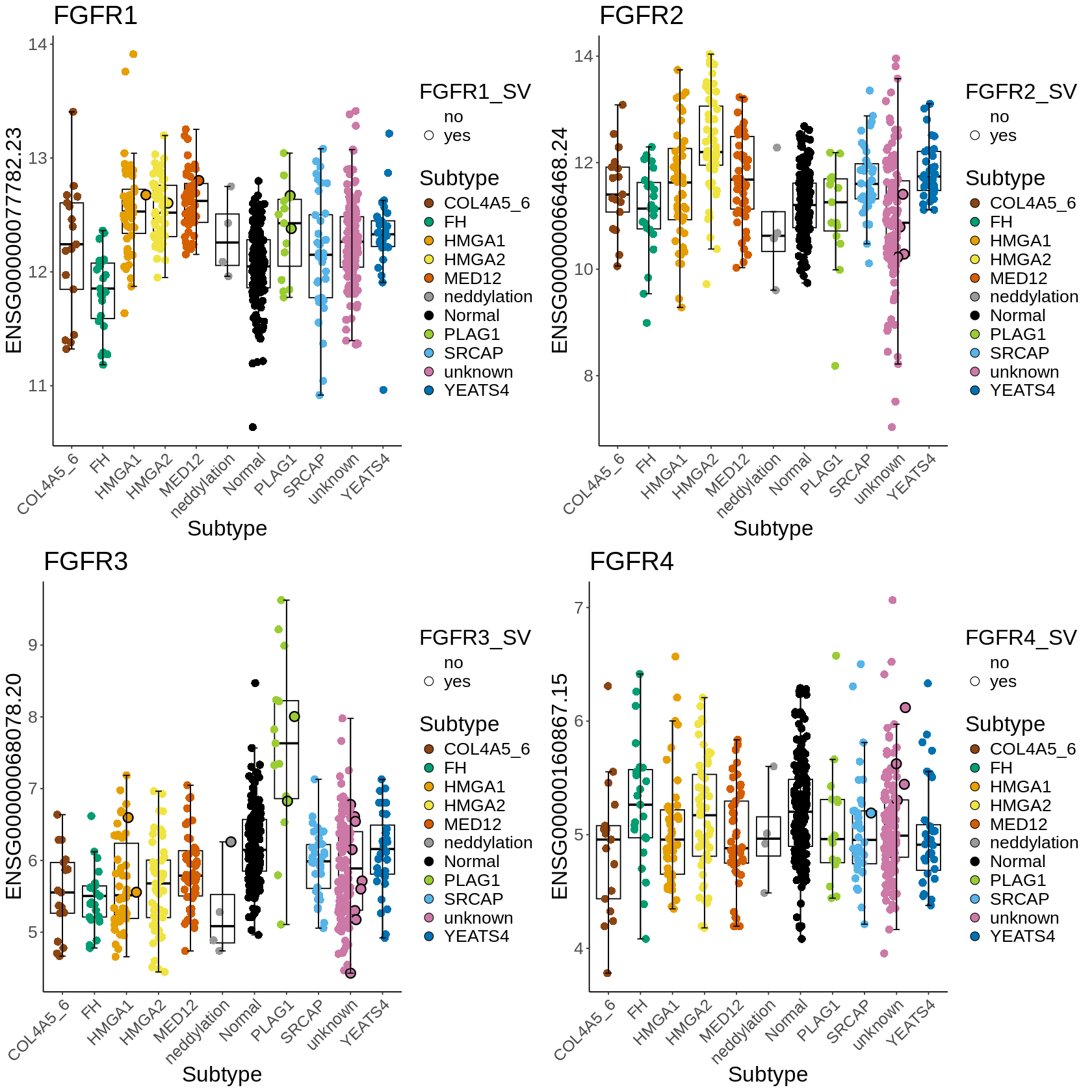
**Figure S2. *FGFR* gene expression levels in ULs with *FGFR* SVs and other UL and myometrium samples.** The UL samples with SVs with a breakpoint at most 1 Mbp from *FGFR* genes are circled with a black line.


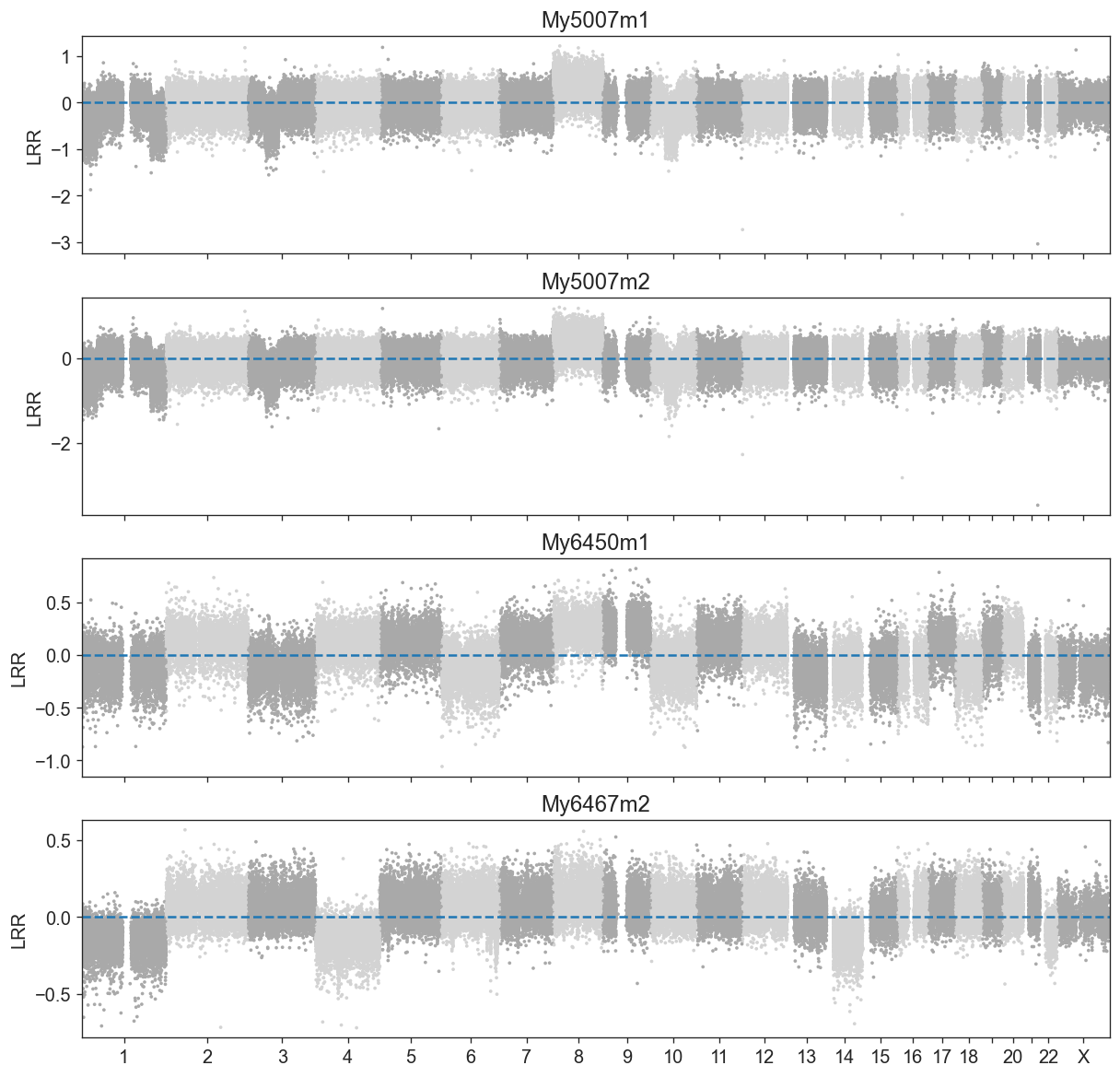


**Figure S3. Somatic whole-chromosome gains of chromosome 8.** Four tumours were identified with chromosome 8 gain resulting in overexpression of *FGFR1*. X-axis shows the reference genome coordinates. The first two tumours harvested from the same patient (My5007) share similar patterns of somatic copy-number aberrations and were determined to be clonally-related. LRR: log R-ratios based on SNP-array data; values above the dashed line (y>0) suggest somatic gain.


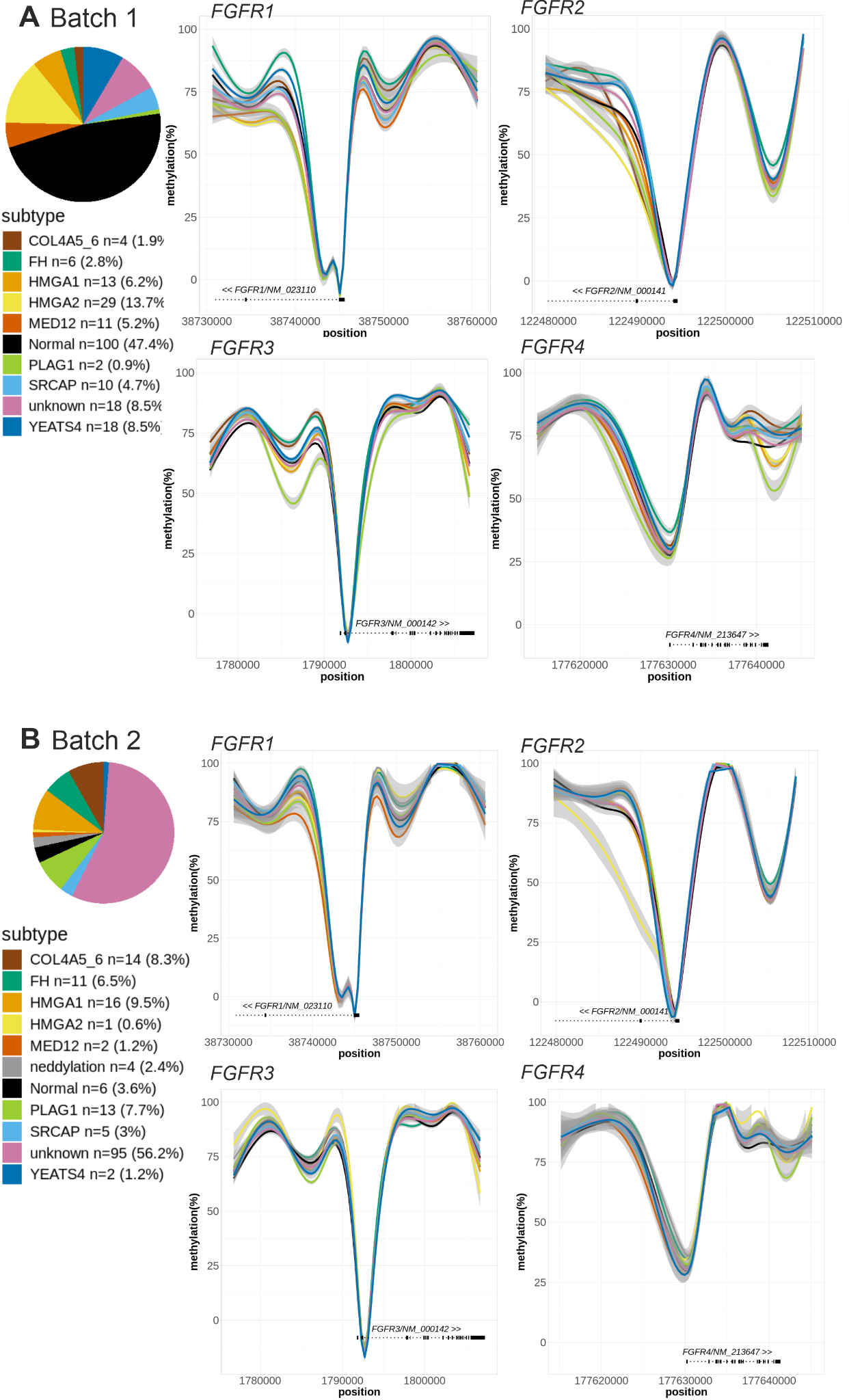


**Figure S4. Methylation levels of *FGFR1-4* in UL subtypes and normal myometrium.** The methylation levels are plotted 15Kbp upstream and downstream from transcription start sites of canonical transcripts. The lines represent smoothing curves based on CpG measurements from different subtypes. Nanopore data was produced in two batches, and the data is displayed separately for **A)** Batch 1 and **B)** Batch 2. HMGA2 ULs show slightly lower methylation levels of *FGFR2*. *FGFR3* overexpression in PLAG1 ULs may be explained by hypomethylation of an upstream region of *FGFR3* in at least a subset of PLAG1 ULs.


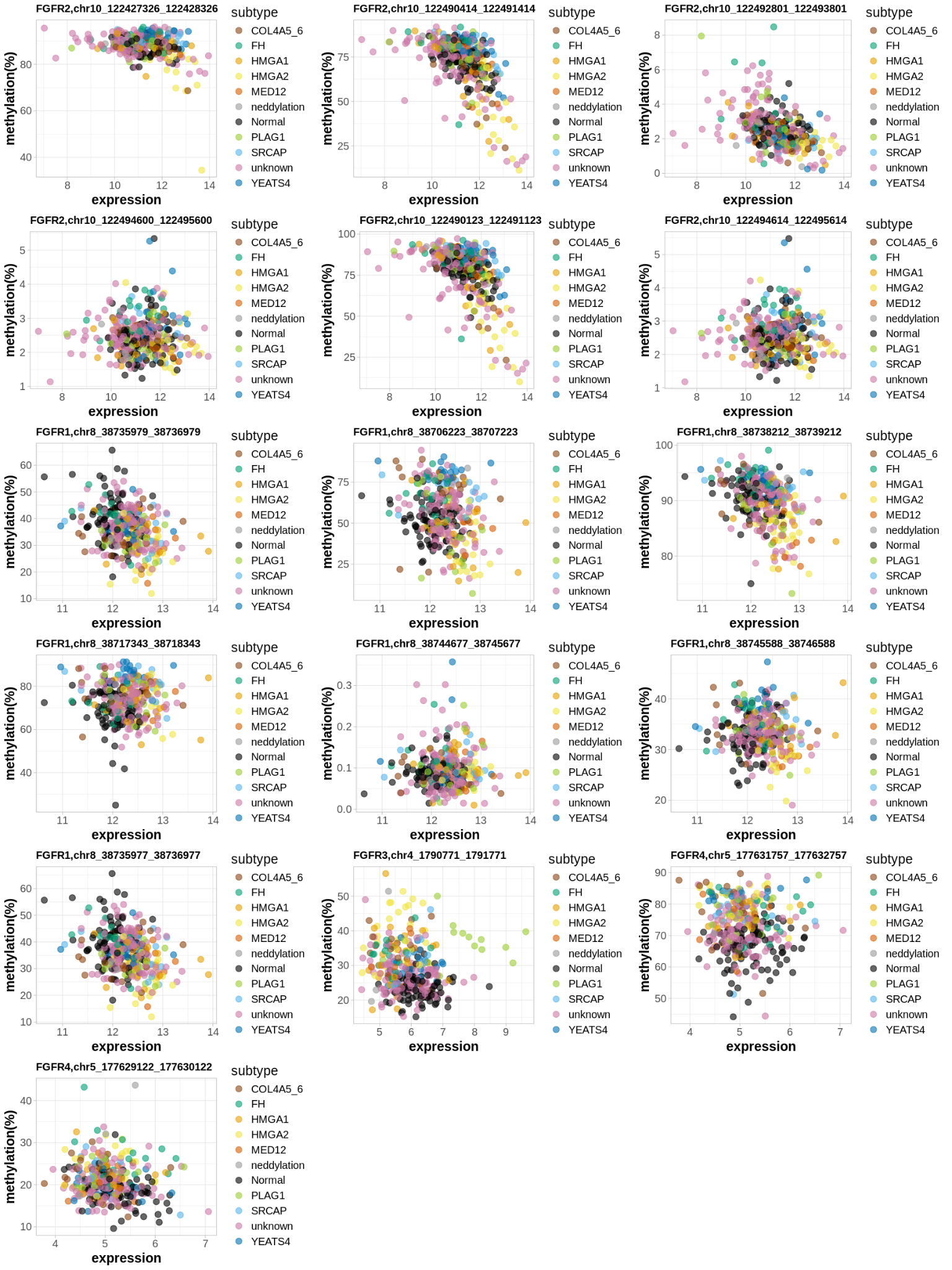


**Figure S5. *FGFR* gene expression level plotted against the average DNA methylation level of promoter regions for each alternative transcript of *FGFR* genes.** The data is shown for all UL and normal myometrium samples with both Nanopore methylation and RNA-seq data available (n=353).

**References**

1 [Berta DG, Kuisma H, Välimäki N, Räisänen M, Jäntti M, Pasanen A *et al.* Deficient H2A.Z deposition is associated with genesis of uterine leiomyoma. *Nature* 2021; **596**. doi:](http://paperpile.com/b/xAjzEJ/ry5nT)[10.1038/s41586-021-03747-1](http://dx.doi.org/10.1038/s41586-021-03747-1)[.](http://paperpile.com/b/xAjzEJ/ry5nT)

2 [Ahvenainen T, Kaukomaa J, Kämpjärvi K, Uimari O, Ahtikoski A, Mäkinen N *et al.* Comparison of 2SC, AKR1B10, and FH Antibodies as Potential Biomarkers for FH-deficient Uterine Leiomyomas. *Am J Surg Pathol* 2022; **46**: 537–546.](http://paperpile.com/b/xAjzEJ/UdYXe)

3 [Mehine M, Ahvenainen T, Khamaiseh S, Härkönen J, Reinikka S, Heikkinen T *et al.* A novel uterine leiomyoma subtype exhibits NRF2 activation and mutations in genes associated with neddylation of the Cullin 3-RING E3 ligase. *Oncogenesis* 2022; **11**: 52.](http://paperpile.com/b/xAjzEJ/HQ9KF)

4 [Mehine M, Kaasinen E, Heinonen HR, Mäkinen N, Kämpjärvi K, Sarvilinna N *et al.* Integrated data analysis reveals uterine leiomyoma subtypes with distinct driver pathways and biomarkers. *Proc Natl Acad Sci U S A* 2016; **113**: 1315–1320.](http://paperpile.com/b/xAjzEJ/pwUUV)

5 [Li H. Minimap2: pairwise alignment for nucleotide sequences. *Bioinformatics* 2018; **34**: 3094–3100.](http://paperpile.com/b/xAjzEJ/MwNS)

6 [Edge P, Bansal V. Longshot enables accurate variant calling in diploid genomes from single-molecule long read sequencing. *Nat Commun* 2019; **10**: 4660.](http://paperpile.com/b/xAjzEJ/MXCa)

7 [Lee SC-H, Burke PJ. NanoStat: An open source, fully wireless potentiostat. *Electrochim Acta* 2022; **422**: 140481.](http://paperpile.com/b/xAjzEJ/CQSF)

8 [De Coster W, Rademakers R. NanoPack2: population-scale evaluation of long-read sequencing data. *Bioinformatics* 2023; **39**. doi:](http://paperpile.com/b/xAjzEJ/wNg7)[10.1093/bioinformatics/btad311](http://dx.doi.org/10.1093/bioinformatics/btad311)[.](http://paperpile.com/b/xAjzEJ/wNg7)

9 [Jokinen V, Mehine M, Reinikka S, Khamaiseh S, Ahvenainen T, Äyräväinen A *et al.* 3’RNA and whole-genome sequencing of archival uterine leiomyomas reveal a tumor subtype with chromosomal rearrangements affecting either HMGA2, HMGA1, or PLAG1. *Genes Chromosomes Cancer* 2023; **62**: 27–38.](http://paperpile.com/b/xAjzEJ/2YQr)
